# Adaptive Model for Magnetic Particle Mapping Using Magnetoelectric Sensors

**DOI:** 10.3390/s22030894

**Published:** 2022-01-24

**Authors:** Ron-Marco Friedrich, Franz Faupel

**Affiliations:** Institute of Materials Science, Faculty of Engineering, Kiel University, 24143 Kiel, Germany; rmfr@tf.uni-kiel.de

**Keywords:** magnetoelectric, magnetic nanoparticle, imaging, inverse problem, blind deconvolution

## Abstract

Imaging of magnetic nanoparticles (MNPs) is of great interest in the medical sciences. By using resonant magnetoelectric sensors, higher harmonic excitations of MNPs can be measured and mapped in space. The proper reconstruction of particle distribution via solving the inverse problem is paramount for any imaging technique. For this, the forward model needs to be modeled accurately. However, depending on the state of the magnetoelectric sensors, the projection axis for the magnetic field may vary and may not be known accurately beforehand. As a result, the projection axis used in the model may be inaccurate, which can result in inaccurate reconstructions and artifact formation. Here, we show an approach for mapping MNPs that includes sources of uncertainty to both select the correct particle distribution and the correct model simultaneously.

## 1. Introduction

Imaging techniques often involve solving inverse problems in order to be able to image the entity of interest sufficiently [1,2]. The interplay between measured data and the model used to invert said data is often neglected, meaning that the model is assumed to reflect reality correctly. However, this poses a source of error for the inversion of the data—the formation of artifacts in the reconstruction due to the use of an incorrect model. To address this problem, techniques were invented that use additional information (a priori knowledge) on the models and source distribution of the inverse problem. One approach that describes the use of additional information on the inverse problem is called Blind Deconvolution, which is used when the impulse response is not exactly known [3,4,5].

Most often, an imaging system can be described via a convolution; hence, the impulse response (or Point-Spread Function) is the quantity that needs to be modeled correctly. Here, the impulse response can be shift-invariant or not, meaning that the shape of the impulse response depends on the position of the source in the underlying distribution. Given the measured data and assumptions on the underlying source distribution, the goal of Blind Deconvolution is to find the correct model that maps the source distribution to the measurement. For this, appropriate subspaces or constraint sets of the source distribution and models have to formulated [3].

Recently, an imaging system for magnetic nanoparticles (MNPs) using magnetoelectric sensors called Magnetic Particle Mapping (MPM) was developed. In MPM, MNPs are excited into the nonlinear magnetic regime using a homogeneous magnetic AC field. The frequency of the excitation field is chosen such that the higher harmonic excitations due to the magnetic nonlinearity coincides with a highly sensitive mechanical resonance of a cantilever-type magnetoelectric (ME) thin film sensor [6]. The magnetoelectric sensor’s magnetic state determines a sensitive axis and is often not exactly known and varies from sensor to sensor [7]. Hence, prior calibration is performed to quantify the sensitive axis such that one can model the system appropriately. However, this axis may change if biasing is applied or the orientation of the sensor is not known exactly [7]. Thus, additional considerations should be taken when modeling the MPM imaging system.

The MPM imaging system can be described as a linear shift-invariant system, where the distribution of MNPs is nonnegative. The impulse response of the system is dependent on the orientation of the MNPs and the sensitive axis of the sensor. These two constraints can be used to create an adaptive scheme that finds the correct model and the underlying MNP distribution simultaneously by using a gradient descent procedure with alternating projections onto feasible sets. The presented approach is also applicable for other systems where projection axes are unknown and may, thus, be adapted for specific imaging systems.

## 2. Materials and Methods

### 2.1. Imaging MNPs

MNPs can be imaged in a variety of ways. MNP imaging techniques that use MNP as tracer material include Magnetic Particle Imaging (MPI) [8,9,10], Scanning Magnetic Particle Spectrometry (SMPS) [11,12,13,14], Magnetorelaxometry Imaging (MRXI) [15,16,17,18] and Magneto Acoustic Tomography (MAT) [19,20,21]. Another type of MNP imaging technique can be categorized into Magnetic Susceptibility Imaging (MSI) [22], one of which is called Susceptibility Magnitude Imaging (SMI) [23]. This technique was further developed using nonlinear MNP responses by spectroscopy AC susceptibility Imaging (sASI) [24] and nonlinear Susceptibility Magnitude Imaging (nSMI) [25].

Approaches for enhanced imaging based on figure of merit optimization for models were performed for several imaging techniques. For example, the authors in [18] performed optimized coil activation sequences in MRXI measurements to reduce the condition number of the model for easier inversion and robustness. Another example includes theoretical enhancements on the SMI setup in [23] as investigated in [26] based on geometric considerations on the figure of merits in inverse problems.

Currently, little investigations have been conducted explicitly with regards to Blind Deconvolution techniques in imaging MNP. In MPI, blind deconvolution techniques were proposed and investigated to address the issue of unknown impulse responses [27]. Most often, prior calibration of imaging systems are sufficient for imaging, yet the possibility for simultaneous characterization of the imaging system and accurate image reconstruction would be an attractive property for any imaging system.

#### Sensing MNPs with Magnetoelectric Sensors

Magnetoelectric sensors were recently used for detecting the magnetic response of MNPs. That this was possible was first shown by [28], where a laminate composite consisting of 500-micrometer-thick PZT with 18-micrometer-thick soft magnetic Ni-based Metglas ribbon. The research was performed in the context of clinical interventions. It was argued that ME sensors could be used for interventions such as sentinel lymph node biopsy (SLNB) for cancer detection, an application that is also argued for other imaging techniques, as mentioned earlier. With their setup, they performed a one dimensional measurement of the magnetic field, which can be thought of as a precursor for imaging MNP with ME sensors. To magnetize MNPs, a permanent magnet was used, and the sensor was aligned such that no in-plane magnetic field component affected the sensor. The smallest amount of MNP they were able to detect was 310 ng at a distance of 2 mm.

ME sensors were also used for the detection of tissue iron content for Biomagnetic Liver Susceptometry (BLS) [29]. In the study, permanent magnets were used to magnetize the sample and bias the sensor simultaneously. The sample is then moved periodically such that a low frequency magnetic signal is created that can be detected via the ME sensor. The source of the magnetic signal was the protein ferritin, for which its magnetic response is paramagnetic. This approach was reinvented in [30] for the imaging of MNP and is called Magnetic Susceptibility Particle Mapping (MSPM). To generate a low frequency signal of the MNP, a motion-modulated approach was taken, where the MNP distribution is mounted on a rotating disc, which is moved through a magnetic field of permanent magnets. The permanent magnets also bias the sensor for high sensitivity, while using the shape anisotropy of the sensor to adjust the extent of biasing.

In the context of this study, some of the just mentioned considerations were used in MPM as well [6]. The shape anisotropy of the sensors will be used for the separation of externally applied field and the MNP field. Imaging of MNP is performed in 2D using AC fields for magnetic excitation and linear stages for translation. Signal acquisition involves an AC field that magnetically excites the MNP to detect higher harmonic responses via the sensor, similar to MPI or sMPS.

The ME sensor only measures a projection of the magnetic field via its the sensitive axis. This axis, in turn, is dependent on the fabrication procedure and magnetic state of the sensor [7]. It is, thus, convenient for an imaging system, which uses ME sensors, to be able to address an unknown sensitive axis while operating in an imaging experiment.

### 2.2. Modeling the MPM Imaging System

In the following, the imaging system for MPM will be derived and important aspects will be highlighted. To model the MPM imaging system, we have to develop a mathematical relationship between the sources (MNPs) and the measurement positions (sensor positions). The MNPs have a magnetic (vector)field associated with them due to their magnetic dipole moment m and the sensor measures only a single projection of the magnetic field via the sensitivity axis s. The next section will deal with the role of the magnetic dipole field and the sensor in context of an imaging system for MNPs. Furthermore, we can expect the system to be linear as the MNPs’ magnetic fields simply superimpose. In the following, lower case bold letters will denote vectors and upper case bold letters will denote matrices. A hat above a vector will denote a vector of unit length. Hence, m and s are the vectors describing the magnetic moment of the MNP and the sensitivity axis of the sensor, respectively. The vector r will denote a spatial position. Vectors $\hat{m}$, $\hat{s}$ and $\hat{r}$ only describe the directions of said quantities. Matrix I is the identity matrix.

The magnetic field $B_{D}$ is given by the following.

(1) $$\begin{array}{cc} B_{D} & =\frac{\mu_{0}m^{⊺}}{4\pi r^{3}}\left(3\hat{r}{\hat{r}}^{⊺}-I\right). \end{array}$$

If we measure only a single projection of the magnetic field via the sensor’s sensitive axis s, we have the following.

(2) $$\begin{array}{cc} \frac{4\pi }{\mu_{0}}B_{D} & =m^{⊺}\frac{\left(3\hat{r}{\hat{r}}^{⊺}-I\right)}{{\left(r^{⊺}r\right)}^{3/2}}s\\ \frac{4\pi }{\mu_{0}∥m∥∥s∥}B_{D} & ={\hat{m}}^{⊺}H\hat{s}. \end{array}$$

The projected magnetic field can, thus, be written in a bilinear form with magnetic moment direction $\hat{m}$, sensitive direction of the sensor $\hat{s}$ and a symmetric matrix H. The functions contained in H and their respective symmetries are important. Due to outer product $\hat{r}{\hat{r}}^{⊺}$, the diagonal will yield symmetric functions while the off-diagonal elements exhibit antisymmetries with respect to the spatial coordinates. This fact will be important at a later stage, because they imply orthogonality of the measurable fields. Only a few correlated fields remain, making estimation of source distributions easier and allowing the possibility of adaptive models in the inverse problem, which will be shown further below.

We now introduce the relationship of the spatial distribution of MNPs and the resulting magnetic fields in space (regions where the field is measured). We denote the spatial magnetic particle distribution as ρ, and the region where particles are present is domain Ω over which they will be integrated. The measurement positions are denoted as $r_{m}$. The resulting magnetic fields $B_{m}$ at the measurement positions $r_{m}$ are then given by the following.

(3) $$\begin{array}{c} \int_{\Omega }\rho \left(r\right)B_{D}\left(r_{m},r\right)d^{3}r=B_{m}\left(r_{m}\right) \end{array}$$

The equation above is a Fredholm integral equation of the first kind [31]. In fact, in this case, the integral equation is a convolution of the following.

(4) $$\begin{array}{c} \int_{\Omega }\rho \left(r\right)B_{D}\left(r_{m}-r\right)d^{3}r=B_{m}\left(r_{m}\right) \end{array}$$

The projected dipole field $B_{D}$ is called the kernel of this equation and is, in this case, equivalent to an impulse response of the system or is also commonly known as the Point-Spread-Function (PSF) used in optics/imaging systems. The mapping from the spatial particle distribution to the measurable signal is the forward model. In this case, the system is linear and shift-invariant, assuming that the Point-Spread-Function does not change depending on the sample position. Linearity stems from the assumption that the particles do not interact with each other (thus not altering the PSF depending on, e.g., local concentrations) and that the magnetic fields linearly superimpose. This assumption can be assumed if homogeneous fields are used, which is to a sufficient degree the case. Therefore, one can describe this system, similar to linear time-invariant (LTI) systems, as a *linear space-invariant* system.

Discretization of Equation (4) yields a system of linear equations, i.e., in the following.

(5) $$\begin{array}{c} \mathrm{Ax}=b \end{array}$$

Here, A denotes the model matrix, which incorporates the orientation of magnetic dipoles and sensor sensitive axis (compare Equation (2)). Vector x is the spatial MNP distribution for which its entries are non-negative (x≥0), and b denotes the superposition of magnetic fields from the MNPs for each measurement position. Here, it is beneficial to explicitly write out the dependence of the model matrix on the magnetic moment direction m and the sensor sensitive axis s.

(6) $$\begin{array}{cc} A= & m_{1}\left(s_{1}A_{11}+s_{2}A_{12}+s_{3}A_{13}\right)\\ + & m_{2}\left(s_{1}A_{21}+s_{2}A_{22}+s_{3}A_{23}\right)\\ + & m_{3}\left(s_{1}A_{31}+s_{2}A_{32}+s_{3}A_{33}\right). \end{array}$$

The model matrix can be more compactly represented by using the Kronecker matrix product (denoted by ⊗):

(7) $$\begin{array}{c} A=\left(m^{T}⊗I_{m\times m}\right)A_{B}\left(s⊗I_{n\times n}\right) \end{array}$$

where $A_{B}$ is the blockmatrix containing all models.

(8) $$\begin{array}{c} A_{B}=\left(\begin{array}{ccc} A_{11} & A_{12} & A_{13}\\ A_{21} & A_{22} & A_{23}\\ A_{31} & A_{32} & A_{33} \end{array}\right) \end{array}$$

Now, given a data vector b, what source distribution x and model A gave rise to the data? This question denotes the Blind Deconvolution problem.

### 2.3. Inverse Problem

Using the forward model we have developed, we now wish to infer the spatial particle distribution from the measured magnetic fields—we are looking for ways to invert the forward model—i.e., we wish to solve the inverse problem. Assuming that the model is accurate, the solution involves computing an estimate that is closely related to the data. For this, we need to minimize the difference between estimate Ax and data b via some form of metric. Commonly chosen is the $L^{2}$ norm (Euclidean norm) as the distance measure between the two vectors Ax and b. This choice stems from the differentiability of this distance, because the norm is induced by the inner product such that an analytic expression can be found. Differentiability is useful because the solutions can be iterated via gradient descent procedures. However, because we are dealing with an inverse problem, the least square minimum is not the ideal choice due to numerical instabilities and amplifications of noise in the measurements [1,2,32].

To combat these issues, one needs to regularize the solution. Regularization refers to the addition of constraints to the original problem that limit the size of the solution vector x—we are looking to both be close to data b and still have physically meaningful results. For this, we add another term to the cost function called the regularizer *R*.

(9) $$\begin{array}{c} \Phi \left(x\right)={∥\mathrm{Ax}-b∥}_{2}^{2}+\lambda R\left(x\right) \end{array}$$

A possible geometric meaning of the regularizer can be imagined as the description of a set in which the solution has to lie. Often, one can show that this description is the same as for constrained optimization via the Lagrange multiplier λ. Thus, the role of the regularization parameter λ is to set the solution size (size as in norm of a vector). There are many types of regularizations, most notably Tikhonov regularization (also called ridge regression) and $L^{1}$ regularization that promotes sparsity in the solution. The latter is also of interest, because $L^{1}$ regularization in combination with a *nonnegativity constraint* sets the total amount of MNP in the system that has to explain the data and, thus, has physical meaning.

**Figure 1:**
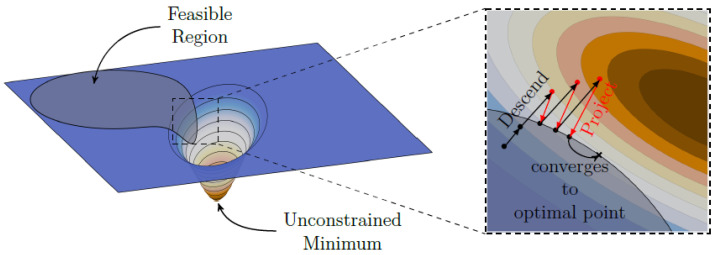
Projected gradient method. Each gradient descent step is projected back into a feasible set. In this manner, the solution becomes regularized, as the solution size cannot lie outside the feasible set. If the feasible set is convex, the solution will converge to an optimal point within the set. Image adapted from [33] (CC BY).

Due to the fact that the $L^{1}$ regularizer is convex but not differentiable, one can employ iterative solutions schemes for solving the inverse problem. A straightforward technique is the projected gradient method (though many different names exists, such as Projected Landweber Iteration [2]). Here, one performs a simple gradient descent step and then projects back into the feasible set defined for the problem, such as projection into the non-negative orthant for the non-negativity constraint and projection onto the $L^{1}$ ball (both can also be described as the projection onto the scaled standard simplex). A visual representation for this procedure can be exemplary observed in Figure 1.

Furthermore, if the magnetic moment direction and sensor sensitive axis also need to be estimated, the objective for solving the inverse problem can be formulated as follows.

(10) $$\begin{array}{c} \min_{x,m,s}\left\{{∥\left(m^{T}⊗I_{m\times m}\right)A_{B}\left(s⊗I_{n\times n}\right)x-b∥}_{2}^{2}+\lambda R\left(x\right)\right\}\;\;\;\;s.t.\;\;\;\;x\geq 0. \end{array}$$

### 2.4. Algorithm

To find the correct spatial MNP distribution as well as the correct model, we propose a two step iterative scheme, which successively updates the MNP distribution and the model of the imaging system via restricting entities to their respective feasible domain. For the MNP distribution, this is mainly performed via the non-negativity constraint, and for the model matrices, only a linear combination of nine models (or rather six due to the symmetries; compare Equation (2)) is allowed, thus defining a subspace of possible models. These restrictions allow for the correct estimation of the MNP distribution and model.

First, an algorithm will be investigated for a system where magnetic moment direction m is perfectly known and sensitive axis s needs to be found in addition to the correct particle distribution, x. Then, the general case for unknown magnetic moment direction m, unknown sensitive axis s and unknown particle distribution x will be investigated and important insights will be highlighted.

#### 2.4.1. Estimating Sensor Sensitive Axis

**Figure 2:**
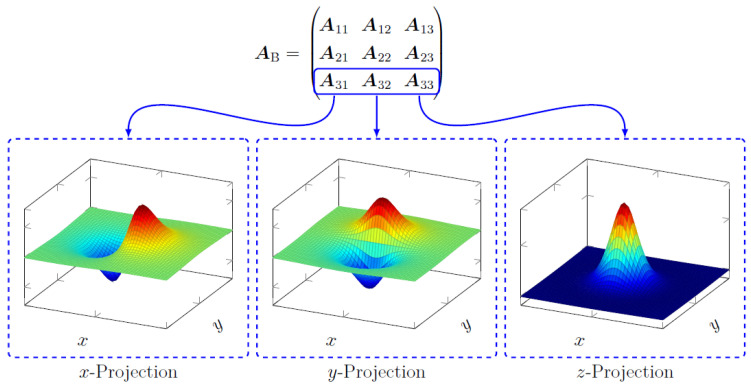
The individual model matrices corresponding to the different combinations of magnetic moment direction and sensitive axis direction refer to different PSFs for the imaging system. In this section, we take the projections for the magnetic moment in the *z* direction, which is indicated by the model matrices enclosed by the blue borders. The dashed boxes show the corresponding PSFs for the model matrices.

In this section, the system for a fixed magnetic moment direction $\hat{m}$ with unknown projection axis $\hat{s}$ from the sensor will be described. We proceed by creating a model matrix that can be described as a superposition of the forward operator in the corresponding axes x,y,z for a fixed magnetic moment direction m in the *z* axis direction, i.e., as shown in Figure 2. We could take any row or column of the block matrix $A_{B}$ to construct a forward operator for the subsequent discussion. The chosen case was taken because it is the projection axis and magnetic moment direction that will be investigated experimentally. Model matrix A can be written for an unknown projection axis as follows.

(11) $$\begin{array}{c} A=s_{1}A_{31}+s_{2}A_{32}+s_{3}A_{33} \end{array}$$

Under these circumstances, the gradient for the cost function can be rewritten as follows:

(12) $$\begin{array}{cc} \nabla_{s}\Phi & =x^{⊺}\frac{\partial A^{⊺}}{\partial s}\left(\mathrm{Ax}-b\right)\\ \; & =D^{⊺}\left(\mathrm{Ds}-b\right) \end{array}$$

with the following.

(13) $$\begin{array}{cc} D & =\left[A_{31}x,A_{32}x,A_{33}x\right] \end{array}$$

(14) $$\begin{array}{cc} s & =\left[s_{1};s_{2};s_{3}\right]. \end{array}$$

As a result, the algorithm to compute particle distribution x and model parameters s can be written as shown in Algorithm 1. The projection operator $P_{+}$ denotes the projection into the non-negative orthant and $P_{C}$ denotes projection onto feasible set *C*. In this case, $P_{C}$ also includes a projection onto the unit sphere, as to denote only the direction of the sensitive axis. As a stopping criterion, the discrepancy principle is used [2].
**Algorithm 1** Model Estimation For Sensitive Axis1:Given: iterations *K*, data b, set of possible model parameters *C*, estimate of noise standard deviation δ, stopping term for discrepancy principle η, projection operator $P_{C}$, $P_{+}$.2:Initialize $A_{31},A_{32},A_{33}$ possible forward operators, particle distribution x, projection estimate s.3:**for** k=1 to *K* **do**4:    $A=s_{1,k}A_{31}+s_{2,k}A_{32}+s_{3,k}A_{33}$.5:    $x_{k+1}=P_{+}\left(x_{k}+{∥A∥}_{2}^{-2}A^{⊺}\left(b-{\mathrm{Ax}}_{k}\right)\right)$6:    $D=\left[A_{31}x_{k+1},A_{32}x_{k+1},A_{33}x_{k+1}\right]$7:    $s_{k+1}=P_{C}\left(s_{k}+{∥D∥}_{2}^{-2}D^{⊺}\left(b-{\mathrm{Ds}}_{k}\right)\right)$8:    **if** ${∥{\mathrm{Ax}}_{k+1}-b∥}_{2}/\delta \leq \eta$ **then**9:        return $x_{k+1},s_{k+1}$10:    **end if**11:**end for**

Under the condition that the correct model can be expressed as the linear combination of model matrices corresponding to the *x*, *y* and *z*-projection of the magnetic field, we write the following cost function.

(15) $$\begin{array}{c} \Phi \left(x\right)=\frac{1}{2}{∥\left(\sum_{i}^{N}s_{i}A_{3i}\right)x-b∥}_{2}^{2}. \end{array}$$

In this case, we would have N=3 matrices for the projections in the *x*, *y* and *z* directions. Recall the dipole functions as depicted in Figure 2. Take, e.g., the orientation of the dipole in *z*-direction and take the product of any two different projections of the dipole fields that correspond to the PSFs in Figure 2. The result will yield equal positive and negative parts (given that we have a fine Cartesian discretization and a spatially large enough domain). This will be important in the following step. If we expand the expression above, we have the following:

(16) $$\begin{array}{c} \Phi \left(x\right)=\frac{1}{2}(x^{⊺}C^{⊺}Cx+b^{⊺}b-b^{⊺}Cx-x^{⊺}C^{⊺}b) \end{array}$$

with $C=\sum_{i}^{N}s_{i}A_{3i}$. Now, the cross terms (i.e., i≠j) in the quadratic forms are equal to zero (considering equidistant sampling of the *x*-*y* plane and that the domain is large enough to capture most magnetic field):

(17) $$\begin{array}{c} x^{⊺}A_{3i}^{⊺}A_{3j}x=0 \end{array}$$

which means that the matrix D is orthogonal.

(18) $$\begin{array}{cc} D & =\left[A_{31}x,A_{32}x,A_{33}x\right]\\ D^{⊺}D & =\mathrm{diag}\left(a\right). \end{array}$$

This follows from the fact that the inner product of the two dipole field projections is zero, since it contains equal amounts of positive and negative parts. We can, thus, obtain the following.

(19) $$\begin{array}{c} \Phi \left(x\right)=\frac{1}{2}\left(1-N\right){∥b∥}_{2}^{2}+\frac{1}{2}\sum_{i}^{N}{∥s_{i}A_{3i}x-b∥}_{2}^{2}. \end{array}$$

Since all terms correspond to strictly convex functions, the problem is uniquely solvable. However, because the number of parameters suffices for any of the matrices $A_{ji}$ to express data b, one will still need to enforce a non-negativity constraint, which will result in the correct estimation in the end. The derivative with respect to the parameters for the projection s is the following:

(20) $$\begin{array}{cc} \nabla_{i}\Phi & =d_{i}^{⊺}\left(d_{i}s_{i}-b\right)\\ \Rightarrow s_{i} & =\frac{d_{i}^{⊺}b}{d_{i}^{⊺}d_{i}^{}} \end{array}$$

(21) $$\begin{array}{cc} s_{i} & =\frac{x^{⊺}A_{3i}^{⊺}b}{x^{⊺}A_{3i}^{⊺}A_{3i}^{}x} \end{array}$$

which means it can be calculated from the projection of the estimate $A_{3i}x$ onto data b. The vector b can thus be expanded by an *N*-dimensional subspace that is constructed from the estimated particle distribution x.

(22) $$\begin{array}{c} b=\sum_{i}^{N}\frac{b^{⊺}A_{3i}x}{∥A_{3i}x∥}\frac{A_{3i}x}{∥A_{3i}x∥}. \end{array}$$

Combining the results, we obtain the following.

(23) $$\begin{array}{cc} \Phi \left(x\right) & =\frac{1}{2}\left(1-N\right){∥b∥}_{2}^{2}+\frac{1}{2}\sum_{i}^{N}{∥\left(\frac{A_{3i}^{}xx_{}^{⊺}A_{3i}^{⊺}}{x_{}^{⊺}A_{3i}^{⊺}A_{3i}^{}x}-I\right)b∥}_{2}^{2}\\ \; & =\frac{1}{2}\left(1-N\right){∥b∥}_{2}^{2}+\frac{N}{2}{∥b∥}_{2}^{2}-\frac{1}{2}\sum_{i}^{N}\frac{{\left(b^{⊺}A_{3i}x\right)}^{2}}{x^{⊺}A_{3i}^{⊺}A_{3i}x}\\ \frac{2}{{∥b∥}_{2}^{2}}\Phi \left(x\right) & =1-\sum_{i}^{N}{\left(\frac{b^{⊺}}{∥b∥}\frac{A_{3i}x}{∥A_{3i}x∥}\right)}^{2}\\ \frac{2}{{∥b∥}_{2}^{2}}\Phi \left(x\right) & =1-\sum_{i}^{N}\cos{\left(\theta_{i}\right)}^{2} \end{array}$$

**Figure 3:**
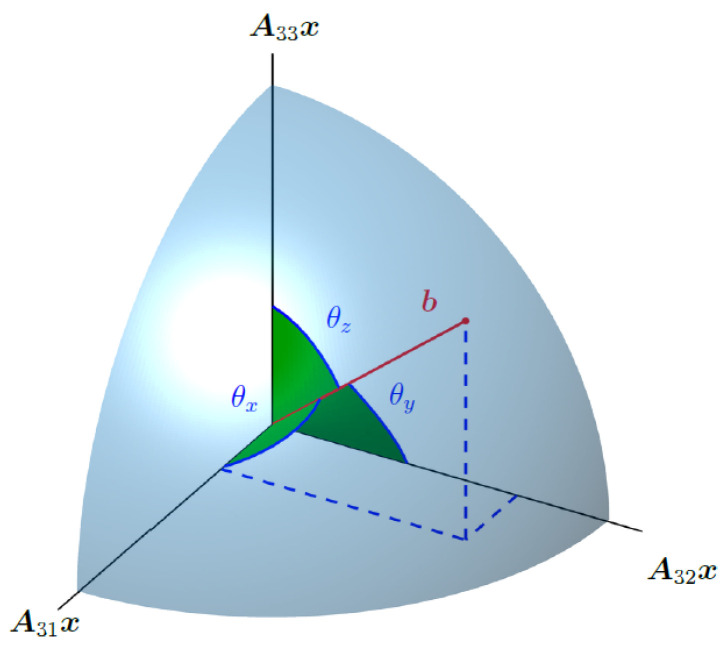
Measurement b is estimated to lie in a 3-dimensional subspace spanned by the estimates for the field projections in the *x*, *y* and *z* directions, i.e., $A_{31}x$, $A_{32}x$ and $A_{33}x$. We, thus, need to find a point on a sphere of radius $∥b∥$.

Here, we see that the cost function is minimized if the projection of estimate $A_{3i}x$ onto data b is maximized (or that the angle $\theta_{i}$ between data b and estimate $A_{3i}x$ is minimized). The direction cosines, thus, add up to 1 if we find the minimum of the cost function. This can be imagined as finding a point on a unit sphere, i.e., the decomposition of $b/∥b∥$ into orthogonal components related to the projection of the magnetic field (see Figure 3). Our task is, thus, finding the very specific subspace that is able to describe data b completely.

#### 2.4.2. Estimating Sensor Sensitive Axis and Magnetic Moment Direction

To estimate both the sensitive axis and magnetic moment direction, they need to be updated within each iteration of the algorithm. For this, the general derivatives with respect to x, s and m need to be computed. The derivatives of the cost function can be written as follows:

(24) $$\begin{array}{cc} \frac{\partial \Phi }{\partial x}= & A^{⊺}\left(Ax-b\right) \end{array}$$

(25) $$\begin{array}{cc} \frac{\partial \Phi }{\partial s}= & M_{s}^{⊺}\left(M_{s}^{}s-b\right) \end{array}$$

(26) $$\begin{array}{cc} \frac{\partial \Phi }{\partial m}= & M_{m}^{⊺}\left(M_{m}^{}m-b\right). \end{array}$$

where $M_{m}$ is given by the following:

(27) $$\begin{array}{c} M_{m}={\left(\left(I_{3\times 3}⊗s^{⊺}\right)\left(\begin{array}{c} M_{1j}^{⊺}\\ M_{2j}^{⊺}\\ M_{3j}^{⊺} \end{array}\right)\right)}^{⊺} \end{array}$$

and $M_{s}$ is given by the following:

(28) $$\begin{array}{c} M_{s}={\left(\left(I_{3\times 3}⊗m^{⊺}\right)\left(\begin{array}{c} M_{i1}^{⊺}\\ M_{i2}^{⊺}\\ M_{i3}^{⊺} \end{array}\right)\right)}^{⊺} \end{array}$$

with the following being the case.

(29) $$\begin{array}{cc} M_{ij}= & \left(\begin{array}{ccc} A_{i1} & A_{i2} & A_{i3} \end{array}\right)\left(I_{3\times 3}⊗x\right)\\ = & \left(\begin{array}{ccc} A_{i1}x & A_{i2}x & A_{i3}x \end{array}\right). \end{array}$$

Important to note in the gradients is the matrix containing all quadratic forms.

(30) $$\left(\begin{array}{c} M_{1j}^{⊺}\\ M_{2j}^{⊺}\\ M_{3j}^{⊺} \end{array}\right){\left(\begin{array}{c} M_{1j}^{⊺}\\ M_{2j}^{⊺}\\ M_{3j}^{⊺} \end{array}\right)}^{⊺}=\left(\begin{array}{ccc} M_{1j}^{⊺}M_{1j} & M_{1j}^{⊺}M_{2j} & M_{1j}^{⊺}M_{3j}\\ M_{2j}^{⊺}M_{1j} & M_{2j}^{⊺}M_{2j} & M_{2j}^{⊺}M_{3j}\\ M_{3j}^{⊺}M_{1j} & M_{3j}^{⊺}M_{2j} & M_{3j}^{⊺}M_{3j} \end{array}\right).$$

**Figure 4:**
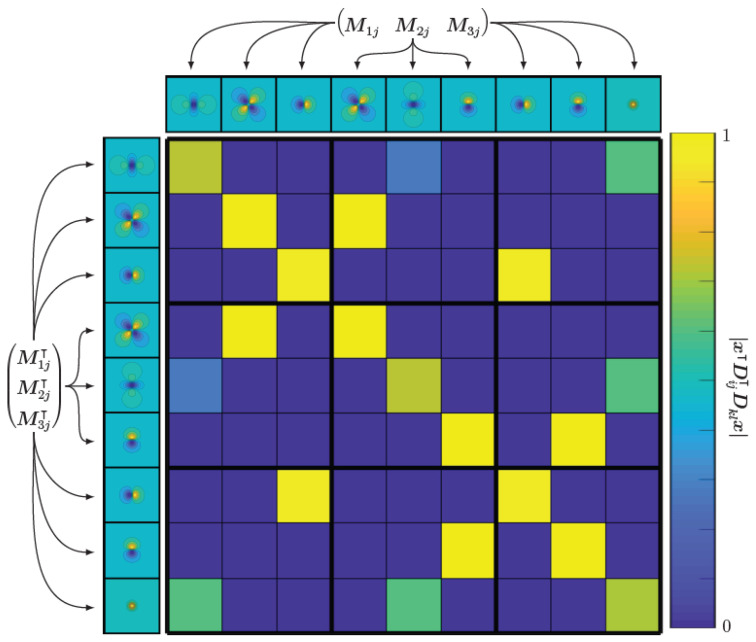
The matrix consisting of all quadratic forms shows the correlation between different magnetic field components for different moment directions. We see that most of the field components are orthogonal to each other, while there are some off-diagonal elements, meaning that there exists a correlation between the corresponding fields. However, they are still linearly independent.

By plotting this matrix, one can gain an idea about the correlation between possible magnetic field components for different dipole orientations, see Figure 4. We see that because we have off-diagonal elements not equal to zero, the fields for different dipole orientations are correlated. On the other hand, we see that the 3×3 block diagonal elements are diagonal sub-matrices, implying that the magnetic dipole fields are orthogonal if the orientation of the dipole lies on the (orthogonal) coordinate system axes. An algorithm that estimates the MNP distribution x, sensitive sensor axis s and magnetic dipole direction m can be seen in Algorithm 2.
**Algorithm 2** Model Estimation1:Given: iterations *K*, data b, estimate of noise standard deviation δ, stopping term for discrepancy principle η, projection operator $P_{+}$, $P_{C}^{\left(1\right)}$, $P_{C}^{\left(2\right)}$.2:Initialize estimate of model parameters s, m, MNP distribution x.3:**for** k=1 to *K* **do**4:    $A=\left(m^{T}⊗I_{m\times m}\right)A_{B}\left(s⊗I_{n\times n}\right)$5:    $x_{k+1}=P_{+}\left(x_{k}-{∥A∥}_{2}^{-2}A^{⊺}\left({\mathrm{Ax}}_{k}-b\right)\right)$6:    $m_{k+1}=P_{C}^{\left(1\right)}\left(m_{k}-{∥M_{m}∥}_{2}^{-2}M_{m}^{⊺}\left(M_{m}m_{k}-b\right)\right)$7:    $s_{k+1}=P_{C}^{\left(2\right)}\left(s_{k}-{∥M_{s}∥}_{2}^{-2}M_{s}^{⊺}\left(M_{s}s_{k}-b\right)\right)$8:    **if** ${∥{\mathrm{Ax}}_{k+1}-b∥}_{2}/\delta \leq \eta$ **then**9:        return $x_{k+1},s_{k+1},m_{k+1}$10:    **end if**11:**end for**

#### 2.4.3. Measurement

**Figure 5:**
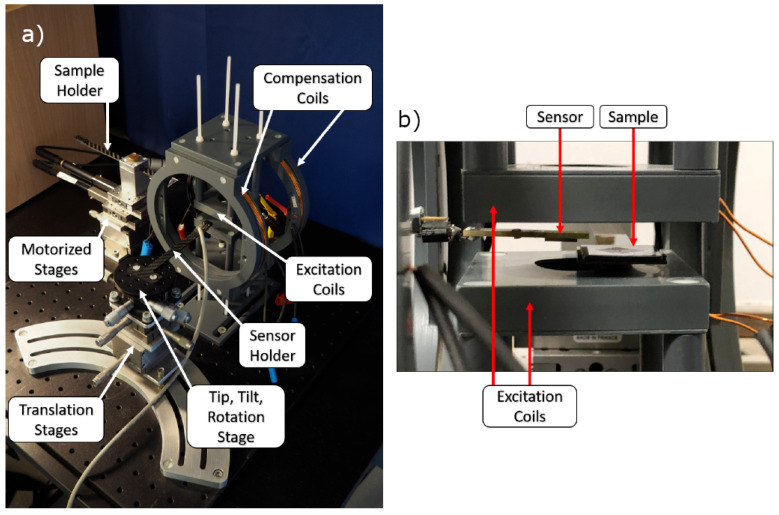
(**a**) Experimental Magnetic Particle Mapping setup. Manual tip, tilt, rotation and translation stage are used to position the sensor with high precision. Motorized stages are used to move the sample with respect to the sensor. (**b**) Close-Up of sensor near the sample between the excitation coils.

The experimental MPM setup can be observed in Figure 5. Shown are the translation stages for position, the sensor and sample and sets of Helmholtz coils for magnetic field generation. One set of Helmholtz coils is used to excite the MNP into the nonlinear magnetic regime, and another set is used for compensation purposes, as will be explained further below. Not shown are the electric appliances, which include an audio amplifier for signal amplification, a charge amplifier for sensor signal amplification and an audio interface to generate the excitation signal and measure the sensor signal. The magnetoelectric sensor used for the experiment is exchange biased [34] in order to avoid an external biasing field, and the fabrication steps can be read up in [6]. The sensor exhibits its first mechanical resonance at about 7.5 kHz. Sensor sensitivity is about 20 kV/T, and the equivalent noise density at resonance is 15 pT/Hz ${}^{0.5}$. The excitation signal is generated by an RME FireFace UC with a sampling frequency of 192 kHz at $1/3f_{r}$ with $f_{r}$ being the resonant frequency of the sensor. The excitation signal is amplified using a PAS2002 audio amplifier and connected to the Helmholtz coils with additional impedance matching. The AC magnetic field generated is about 10 mT. The sensor is aligned using the manual tip, tilt, rotation and translation stages such that its shape anisotropy is used to attenuate some of the influence of the applied excitation field. Additionally, another magnetic AC field is applied with low amplitude at sensor resonance, for which its amplitude and phase are tuned to destructively interfere with the background signal. Then, the sample containing MNP can be inserted and measured. For measurements, equidistant points (40×40) are sampled in space, which correspond to an area of 20×20 mm${}^{2}$, and a signal is measured with a sample rate of 32 kHz and a frame size of 4096 samples, yielding a spectral resolution of 7.8 Hz. In the spectrum, phase and amplitude at resonance are captured, which should ideally only contain the responses to MNPs.

**Figure 6:**
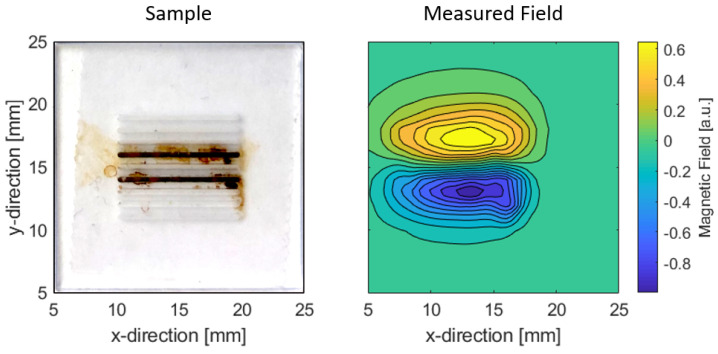
(**Left**): image of the sample with MNP in trenches. (**Right**): measured magnetic field of the sample.

An image of the MPM sample and the measured magnetic field can be seen in Figure 6. For this, MNP CT100s (fluidMAG, Chemicell, Berlin, Germany) were placed into parallel trenches of a sample holder. The sample has an area of $20\times 20\;{\mathrm{mm}}^{2}$. The trenches are 0.5 mm deep and have a length of 1 mm. The filled trenches are 3 mm apart. The total amount of MNP roughly amounts to 300 μg. The magnetoelectric sensor was placed at a distance of circa 2 mm above the sample. Additionally, the associated measured magnetic field can be seen next to it. There exists a translational offset in the origins of both images; hence, the field is not directly above the trenches in direct comparison. This was no influence on the reconstruction.

## 3. Results and Discussion

### 3.1. Simulation

In the following section, two cases of the blind deconvolution algorithm are investigated, which are listed in Table 1 indicated as Case I and Case II. The cases correspond to the unknown parameters in the model matrix A, i.e., sensitive axis s and magnetic moment direction m. These parameters are either known or unknown and, hence, need to be estimated. Case I for s known and m is unknown (∘), and vice versa (×) they are equivalent due to the bilinear relationship in Equation (2). Case 0 refers to the normal deconvolution when the model is correctly known and will not be treated. The simulations are performed without noise for a maximum number of 500 iterations if not stated otherwise.

#### 3.1.1. Case I

In the following example, an unknown sensitive axis of the sensor is taken and the magnetic moment direction is known. Even though the cost function is strictly convex (refer to Equation (19)), the reconstruction for any model combination (set of $s_{i}$ in Equation (19)) could explain the data. What is important is then the non-negativity constraint, such that it acts as a guide to find the correct model. For the case where the magnetic moment direction is known and the sensitive axis has to be estimated while computing the MNP distribution, Algorithm 1 will be used. For each MNP distribution update, the estimated sensitive axis is updated.

**Figure 7:**
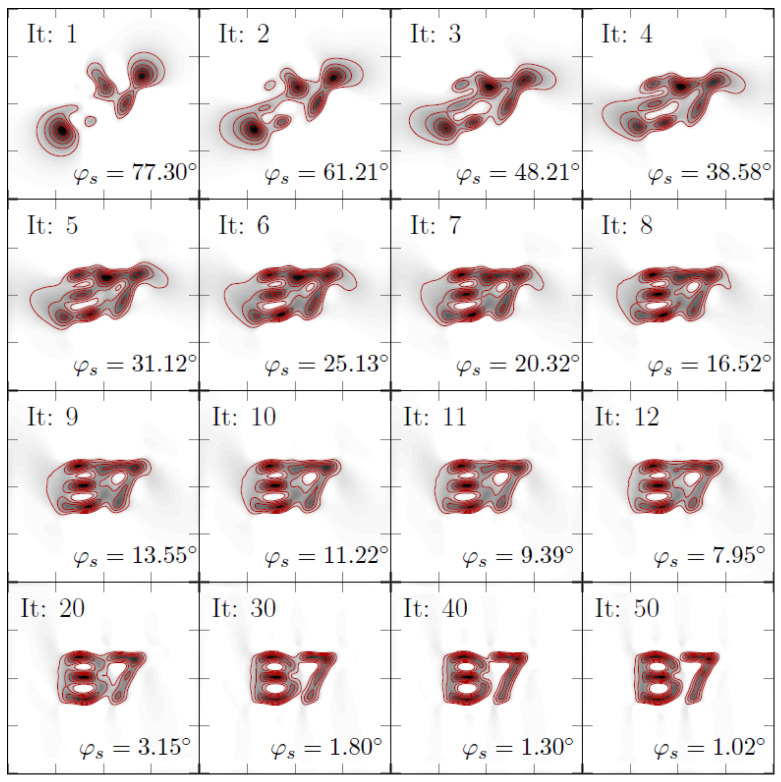
Adaptive reconstruction to iterate the MNP distribution x and the sensitive axis s simultaneously. Shown are individual iterations as denoted in the upper left corner of each subplot. In the lower right corner of each subplot, the estimated sensitive axis direction (polar) is shown. The ground truth for this axis has an angle of $\varphi_{m}=0^{\circ }$. The black color indicates MNPs, and the red lines are level sets to guide the eye. Dimensions of each square correspond to an area of 20×20 mm${}^{2}$.

An overview of the iterations of the inversion can be seen in Figure 7. As ground truth, the letters “B7” were used. The dimensions were $20\times 20\;\;{\mathrm{mm}}^{2}$ with 50×50 equidistant points at a *z*-distance of 1 mm. The data for the reconstruction were computed via a sensitive axis that lies in the *x*-*y* plane at an angle of $0^{\circ }$ (*x*-direction). The algorithm is initiated via a sensitive axis direction of $90^{\circ }$. It can be observed that the angle approaches a value of $1^{\circ }$ for the 50th iteration and that the MNP reconstruction yields the letters “B7”, which indicates that the algorithm is able to simultaneously find the MNP distribution and the sensitive axis direction.

**Figure 8:**
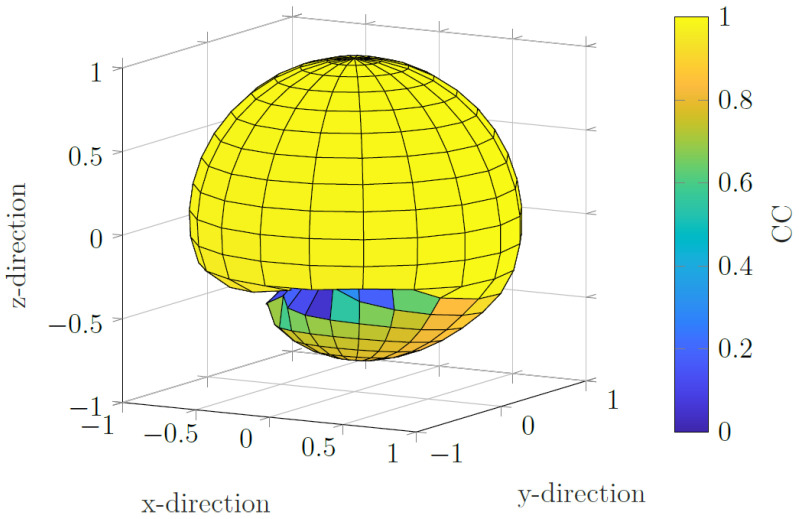
Correlation coefficient for different initial sensitive axis directions. The true sensitive axis lies in the *x* direction. For the upper half space (z≥0), the correlation coefficients form a sphere, indicating that the algorithm converges to the true MNP distribution. For the lower half plane, there are regions where the right MNP distribution is not found via the algorithm.

To further investigate the algorithm, a true projection axis for the data is chosen in the *x*-direction. The algorithm is initiated using a projection axis for all spatial directions that lie on the unit sphere. To quantify the ability to correctly estimate the MNP distribution, the correlation coefficient is taken of the final iteration. Pearson’s correlation coefficient (CC) acts as a measure of spatial accuracy of the reconstructed particle distribution compared to the ground truth. The absolute value of the correlation coefficient from the MNP reconstruction for the *initial* sensitive axis direction is used as a radius for that direction. In the case of the correct estimation of the projection axis for all initial directions, the result would be a sphere. Figure 8 shows the results. It can be seen that, for the initial sensitive axes in the upper half plane (z≥0), the algorithm converges to the correct MNP distribution, indicated by the large correlation coefficient. For some regions in the lower half plane, the algorithm fails to converge to the correct MNP distribution. However, if a good guess is taken for the true sensitive axis, the algorithm will simultaneously find the sensitive axis and the MNP distribution.

#### 3.1.2. Case II

Next, the case when both the projection axis and the magnetic moment orientation are unknown is considered. In this case, the estimation procedure involves updating the particle distribution, followed by an update of the projection axis of the sensor and followed by an update on the magnetic moment direction. Whether the projection axis or the magnetic moment direction is updated first is irrelevant, because there exists an ambiguity between the magnetic moment direction and the projection axis, as is apparent from the bilinear form that dictates the impulse response of the system and the fact that the matrix of the bilinear form is symmetric (refer to Equation (2)).

To investigate the reliability of the proposed algorithm (see Algorithm 2), one sweeps the parameter space for all orientations of the projection axis and magnetic moment direction. We chose the correct (i.e., belonging to the model that gave rise to the data) projection axis and magnetic moment direction in the *x* and *z* directions, respectively. In addition, a box constraint on the projection axis s and magnetic moment direction m in the form of a predetermined half-space is imposed. That is, the *x* component of the projection axis cannot be negative, and the *z* component of the magnetic moment direction cannot be negative. Furthermore, it is imposed that the projection axis and magnetic moment direction is of unit length, which is implemented via projection operators $P_{C}^{\left(1\right)}$ and $P_{C}^{\left(2\right)}$. A non-negativity constraint on the particle distribution x is imposed as well.

**Figure 9:**
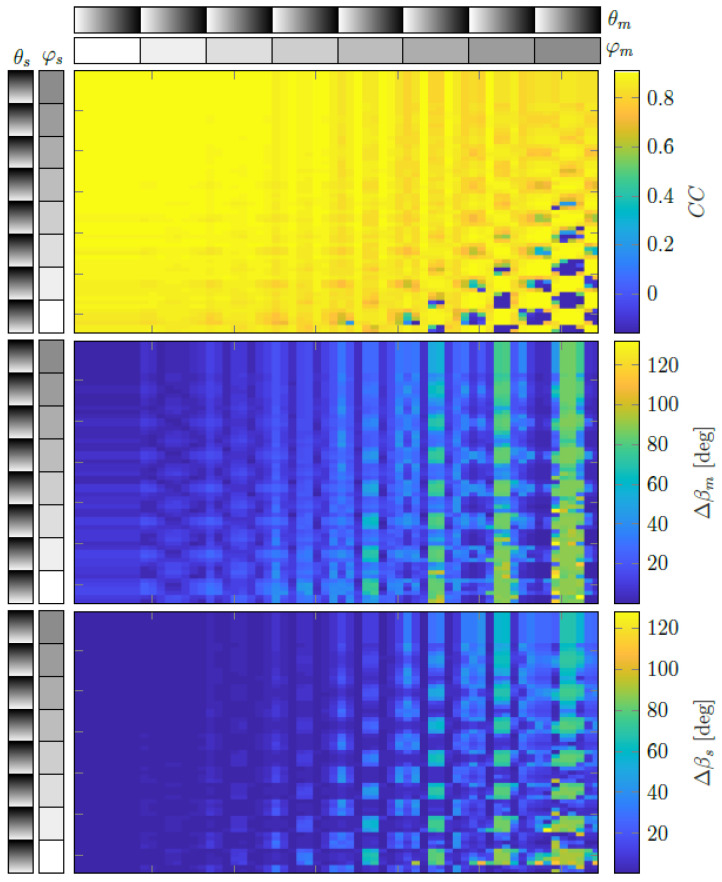
(**Top**): Correlation coefficient of the reconstruction to the ground truth. (**Middle**): Angle error of the magnetic dipole direction. (**Bottom**): Angle error of the sensitive axis direction. All points in the graph are results for a different combination of an initial dipole field direction, indicated by angles $\theta_{m}$ (polar) and $\varphi_{m}$ (elevation), and sensitive axis directions, indicated by angles $\theta_{s}$ (polar) and $\varphi_{s}$ (elevation). The grey colorbars denote the angle (white is $0^{\circ }$ and black is $360^{\circ }$).

In Figure 9, one can observe the correlation coefficient of the estimated MNP distribution to the ground truth as well as the angle differences of the estimated projection axis of the sensor and the angle difference of the estimated magnetic moment direction (with respect to the ground truths, respectively). It can be seen that correlation coefficient CC is high for most parameter combinations of the directions of sensitive axis s and dipole orientation m, as indicated by their direction angles in spherical coordinates. For the dipole moment m, angles $\theta_{m}$ (polar) and $\varphi_{m}$ (elevation) describe the direction, and for the sensitive axis s, angles $\theta_{s}$ (polar) and $\varphi_{s}$ (elevation) describe the direction. Furthermore, it can also be seen that, for initial parameter combinations where the correlation coefficient is large, the angles between the true and estimated directions of s and m are small. This indicates that the algorithm is capable of finding the correct MNP distribution while also estimating the directions of s and m correctly.

### 3.2. Experiment

#### Reconstruction

**Figure 10:**
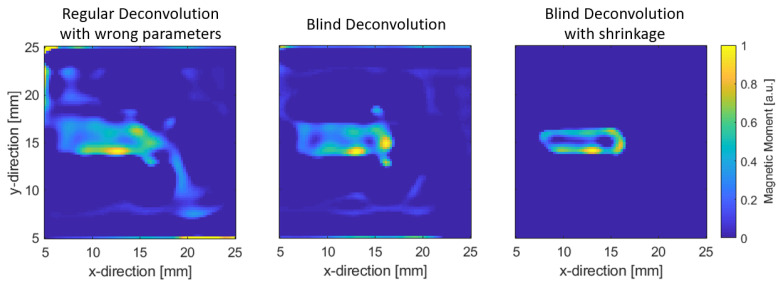
(**Left**): regular deconvolution with sensitive axis off by $20^{\circ }$; (**middle**): Blind Deconvolution as outlined in Algorithm 2; (**right**): Blind Deconvolution with additional shrinkage by soft thresholding.

The reconstruction is performed via using an orientation of the sensitive axis that is $20^{\circ }$ off the true axis. Not knowing this prior to measurement results in the formation of artifacts in the reconstruction, as can be seen in Figure 10. In direct comparison, the reconstruction using Algorithm 2 results in the reconstruction having significantly less artifacts, being more localized and having a better resolution, because the trenches can roughly be imaged individually. We suspect that the sensor geometry has to be considered in the model for a more accurate reconstruction and better resolution. As of now, the sensor is regarded as point-like. A discussion of the notion of resolution in inverse problems can be found in Appendix A. Additionally, for further enhancement on the image, since fringes are still present around the reconstruction, shrinkage (soft thresholding) can be applied via a projection onto the scaled $L^{1}$ ball in addition to the projection into the non-negative orthant. As can be seen from the Figure, fringes are suppressed, and a clearer reconstruction is formed. The choice of the magnitude of the $L^{1}$ ball is not chosen arbitrarily but can be roughly estimated from the measurement itself. The discussion on this is shown in Appendix B.

## 4. Conclusions

It was shown that the proposed adaptive inversion scheme is able to estimate both the model parameters and MNP distribution simultaneously. The approach shown is able to overcome unknown initial information, such as sensor sensitivity direction, and estimate it correctly. Thus, the generalized model can be used in circumstances where the sensor sensitive axis is not known exactly or incorrectly measured; thereby, it reduces sources of error in the model for better reconstructions, which ultimately improves imaging applications.

## Figures and Tables

**Table 1:** Investigated cases.

	*s* Known	*s* Unknown
m known	Case 0	Case I
m unknown	Case I	Case II

**Figure A1:**
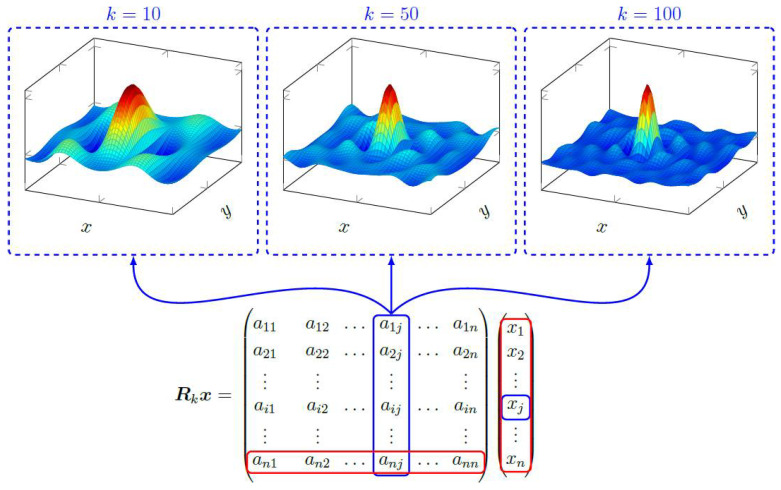
The columns of the resolution matrix $R_{k}$ reflects the spread of a delta-like input, which can be thought of as the Point-Spread Function of the system. For the inverse operator for different iterations *k*, the resolution matrix attains a more delta-like response, which means that the spread is reduced and that resolution is enhanced.

## Data Availability

The data that support the findings of this study are available from the corresponding author upon request.

## Abbreviations

The following abbreviations are used in this manuscript:

MPM	Magnetic Particle Mapping;
MNP	Magnetic Nanoparticle;
PSF	Point-Spread-Function.

## Appendix A. On Notion of Resolution in Inverse Problems

Varieties in approaches of solving inverse problems can be roughly categorized into two types: linear and nonlinear inversion schemes. This refers to the mapping of the measurement data back to the underlying distribution that gave rise to the data. If the inversion is linear, a linear inversion operator can be constructed (i.e., a matrix), and the inversion is independent on the distribution (e.g., MNP) and, hence, depends on the model only. The construction of an inverse operator is only performed to an approximate degree—no full inverse operator will be applied since overfitting would result in erroneous reconstructions that do not reflect reality. Here, statements can be made about the effectiveness of the imaging technique on a general basis. A nonlinear inversion scheme is dependent on the underlying distribution; hence, no general statements can be made. In the following, these statements will be elucidated more in depth.

Resolution in an imaging system refers to its ability to resolve features that are spatially close. The resolution for an imaging system such as a microscope normally uses the Point-Spread Function as a figure of merit. This quantity reflects the spatial spread of a point source input to the imaging system. With the use of deconvolution techniques, the resolution can be enhanced, and the spread of the Point-Spread Function can be reduced.

The proper assessment of the enhancement of a linear imaging system can be performed via the so-called resolution matrix R [35,36]. The meaning of this operator is that no features can be reconstructed that are sharper than given by the columns, which in turn makes using inverse operators’ intrinsic resolution limited [37,38]. To introduce this matrix, we start off with the system of equations that relate particle distribution x with magnetic field b via model matrix A.

(A1) $$\begin{array}{c} \mathrm{Ax}=b. \end{array}$$

The approach for solving this system of equations uses an inverse operator that, when applied to the left hand side of the equation, yields just the particle distribution x.

(A2) $$\begin{array}{c} A^{†}\mathrm{Ax}=x. \end{array}$$

Here, $A^{†}$ denotes the pseudoinverse of model matrix A. However, under normal circumstances, only an approximate inverse operator is used, which we will denote as $A_{k}^{†}$. Subscript *k* refers to an iteration number for different approaches of computing the pseudoinverse, which will be shown further below. The reason that only an approximate inverse operator is used is that, otherwise, noise would be amplified in the measurement vector b, which would yield unreliable/unphysical results for the particle distribution x. We can, thus, write the following.

(A3) $$\begin{array}{c} A_{k}^{†}\mathrm{Ax}=A_{k}^{†}b\\ R_{k}x=A_{k}^{†}b. \end{array}$$

Operator $R_{k}=A_{k}^{†}A$ refers to the resolution matrix for a given approximate pseudoinverse. To compute the resolution matrix, we choose two different approaches: the singular value decomposition (SVD) and gradient descent (GD). The resolution matrices associated with these approaches are as follows.

(A4) $$\begin{array}{cc} R_{\mathrm{GD},k}= & \alpha V\left(\sum_{n=0}^{k-1}{\left(I-\alpha S^{2}\right)}^{n}\right)S^{2}V^{⊺} \end{array}$$

(A5) $$\begin{array}{cc} R_{\mathrm{SVD},k}= & V_{k}S_{k}^{-1}U_{k}^{⊺}A \end{array}$$

Here, $A={\mathrm{USV}}^{⊺}$, $\alpha ={∥A^{⊺}A∥}^{-1}$ and subscript *k* either refers to the number of iterations for gradient descent or the number of left and right singular vectors corresponding to the first *k* singular values (i.e., such that $A\approx U_{k}S_{k}V_{k}^{⊺}$).

Let us look into the meaning of these matrices in more detail. The resolution matrix can be thought of in two ways:

- The columns can be understood as the spatial spread of a delta-like input, i.e., the Point-Spread Function.
- The rows can be understood as the convolution of the MNP distribution with the Point-Spread Function. It is the (scaled) averaging function of the system.

As observed in Figure A1, the columns (blue enclosure of the matrix) represent the spatial spread of a delta-like input at particle position *j*. The red enclosure in the matrix refers to the weighted (the weights can be negative) sum of all field contributions from all MNP, such that it refers to an *averaging function* of the MNP responses. Due to the fact that the system is a linear space-invariant system, the resolution is the same for all positions. *Resolution* could heuristically be chosen to be the full width half maximum of the peak of the columns. It has to be noted that the resolution may be spatially anisotropic depending on the shape of the impulse response of the imaging system.

The peaks as shown in Figure A1 become narrower with increasing iteration numbers. This means that the resulting overall resolution is given by the last iteration we were able to perform without fitting noise. For this, there are several criteria that can be used for the termination of the reconstruction [2]. It has to be emphasized that the resolution matrix relies on the analytic expressions presented here such that procedures such as the proximal gradient algorithm for the $L^{1}$ projection cannot meaningfully produce a resolution matrix. The only cases where an analytic expression can be found is if the regularizer is differentiable (e.g., Tikhonov regularization), semiconvergent properties of gradient descent are used [2] or truncation via a singular value decomposition is applied.

A more general perspective on the resolution matrix (or rather, resolution map) is that it can be viewed as the effect of a single input on the vector b, which subsequently needs to be inverted [39]. For this, we look at the change of reconstruction $\delta x_{i}$ from unit change ${\hat{e}}_{i}$ in x via the inversion operation G.

(A6) $$\begin{array}{cc} \delta x_{i} & =G\left(A\left(x+{\hat{e}}_{i}\right)\right)-G\left(\mathrm{Ax}\right) \end{array}$$

In case of linearity, that is, if an analytic expression is found via an approximate (generalized) inverse $A_{k}^{†}$, then we have the following.

(A7) $$\begin{array}{cc} \delta x_{i} & =G\left(A\left(x+{\hat{e}}_{i}\right)\right)-G\left(\mathrm{Ax}\right)\\ \; & =A_{k}^{†}A{\hat{e}}_{i}+A_{k}^{†}\mathrm{Ax}-A_{k}^{†}\mathrm{Ax}\\ \; & =R_{k}{\hat{e}}_{i}. \end{array}$$

Thus, in the linear case, output $\delta x_{i}$ from a unit change directly yields a column of the resolution matrix, which tells us about the Point-Spread Function/spatial spread of the parameter $x_{i}$ of particle distribution x. Important to point out is that this inversion process is only dependent on the model itself and not on the particle distribution. This is a direct result of the linearity of the inversion process.

On the other hand, if the inverse map does not obey linearity, which is the case for, e.g., $L^{1}$ regularization, we have the following.

(A8) $$\begin{array}{c} G\left(A(x+{\hat{e}}_{i})\right)\neq G\left(\mathrm{Ax}\right)+G\left(A{\hat{e}}_{i}\right). \end{array}$$

As a result, spatial spread $\delta x_{i}$ of parameter $x_{i}$ depends on particle distribution x itself, and a general statement of the resolution for a model inversion procedure, when the inverse map is not linear, cannot be given. Therefore, assessment of resolution is in this case is only beneficial if a standard reference is used such that the results can be comparable.

## Appendix B. Estimation of Magnetic Content

In the following, it will be shown that the magnetic content of an imaging experiment can be estimated from the measurement alone. For this, we consider a measurement in an *x*–*y* plane with equidistant measurement positions. We further set the magnetic moment direction into the *z* direction and sensitive axis direction in the *x* direction. The MNP distribution is distributed in an *x*–*y* plane below the measurement plane. We, thus, write for magnetic moment m as follows:

(A9) $$\begin{array}{cc} m & =∥m∥\left(\begin{array}{c} 0\\ 0\\ 1 \end{array}\right) \end{array}$$

and for the sensitive axis s, we have the following.

(A10) $$\begin{array}{cc} s & =∥s∥\left(\begin{array}{c} 1\\ 0\\ 0 \end{array}\right) \end{array}$$

The associated magnetic field of a point dipole is then the following.

(A11) $$\begin{array}{c} B_{x}=\frac{\mu_{0}∥m∥∥s∥}{4\pi }\frac{3xz}{{\left(x^{2}+y^{2}+z^{2}\right)}^{5/2}}. \end{array}$$

The projection above is antisymmetric with respect to the *x* direction. Integration of this projection in the x-direction yields the following.

(A12) $$\begin{array}{c} \int_{-\infty }^{x}B_{x}dx=-\frac{\mu_{0}∥m∥∥s∥}{4\pi }\frac{z}{{\left(x^{2}+y^{2}+z^{2}\right)}^{3/2}}. \end{array}$$

The function is of equal sign from −∞ to +∞ for the *x* and *y* variables. The next step involves integrating over the whole x−y plane. The result is the following.

(A13) $$\begin{array}{c} \int_{-\infty }^{\infty }\int_{-\infty }^{\infty }-\frac{\mu_{0}∥m∥∥s∥}{4\pi }\frac{z}{{\left(x^{2}+y^{2}+z^{2}\right)}^{3/2}}dxdy=-\frac{\mu_{0}∥m∥∥s∥}{2}. \end{array}$$

Thus, the given procedure is proportional to the magnetic moment, which is, mathematically speaking, the $L^{1}$ norm of the negative integrated dipole field projection. Therefore, one can calculate the magnetic moment directly from the data, given that a sufficiently large region of the magnetic field is measured to approximate the procedure given above. It is important to point out that the result is independent of the *z*-distance. On one hand, this means that one can estimate the magnetic moment without knowing the *z*-distance, while on the other hand it means that there is no depth information from this procedure. To generalize this approach, given an arbitrary spatial distribution of magnetic moments and given that the measured projection is equal to the example given above, we denote the magnetic field of a magnetic moment as $B_{D}$. We can write the magnetic field associated with a moment distribution, given as $B_{L}$, as follows.

(A14) $$\begin{array}{cc} B_{L}\left(r\right) & =\int_{\Omega }B_{D}\left(r-r^{\prime },m\right)\rho \left(r^{\prime }\right)d^{3}r^{\prime }. \end{array}$$

As above, we assume the same direction of magnetic moment m and integrate in the *x*-direction and then integrate over the x−y plane. Since the integral is not dependent on the *z*-distance (as explained above), one obtains the following result.

(A15) $$\begin{array}{c} -\frac{2}{\mu_{0}∥m∥∥s∥}\int_{-\infty }^{\infty }\int_{-\infty }^{\infty }\int_{-\infty }^{x}B_{L}\left(r\right)dxdxdy=\int_{\Omega }\rho \left(r^{\prime }\right)d^{3}r^{\prime }. \end{array}$$

Since moment distribution ρ is non-negative (i.e., ρ≥0), the following statement is obvious.

(A16) $$\begin{array}{c} {∥\rho ∥}_{1}=\int_{\Omega }\rho \left(r^{\prime }\right)d^{3}r^{\prime }. \end{array}$$

Thus, the total amount of magnetic content can be computed from the magnetic field if the measured projection is the same as in the form above. It would also work for the projection in the *y* direction. In this way, one can infer the total amount of particles from the measurement itself and can use this for regularization via projection onto the scaled $L^{1}$ ball in the projected gradient method. One has to point out that, because the result is independent on the *z* distance, any reconstruction from *any* height that is able to construct vector b via estimate Ax yields the same amount of MNP content.
